# From Betrayal to Meaning: A Fictional Case Vignette Illustrating Existential Recovery After Complex Trauma

**DOI:** 10.1192/j.eurpsy.2026.11876

**Published:** 2026-07-31

**Authors:** A. Melo, C. Oom, V. Medeiros, F. Moutinho, G. Jesus

**Affiliations:** 1 Hospital Vila Franca de Xira, Vila Franca de Xira, Portugal; 2 Psychiatry, Hospital Vila Franca de Xira, Vila Franca de Xira, Portugal

## Abstract

**Introduction:**

Fictional narratives can be powerful educational tools in psychiatry, allowing exploration of sensitive themes without breaching confidentiality. We present “Ash,” a fictional character whose developmental history and psychological trajectory mirror common clinical patterns of complex trauma, depressive collapse and existential recovery.

**Objectives:**

Our objective was to demonstrate how a detailed fictional case vignette can serve as an innovative teaching tool in psychiatry, illustrating the psychodynamic and existential processes involved in complex trauma, depressive collapse and recovery of meaning.

**Methods:**

We developed a detailed fictional case vignette drawing on psychoanalytic, existential and phenomenological concepts and analysed Ash’s life history, emotional landscape and adaptation to illustrate key psychodynamic mechanisms and potential teaching applications for psychiatry trainees.

**Results:**

Ash, once a devoted and perfectionistic youth, discovers he was betrayed and effectively “sold” to an imperial institution. This revelation precipitates a severe depressive episode characterised by anhedonia, loss of purpose and existential despair. Over time he shifts from striving for external validation to cultivating curiosity, endurance and authentic presence.

The analysis identified three interconnected processes: (1) betrayal and early attachment trauma leading to perfectionism and over-investment in an institution; (2) depressive collapse and existential despair following discovery of betrayal; and (3) gradual reconstruction of meaning and authentic identity through curiosity and acceptance. These processes parallel patterns observed in real clinical settings and provide a framework for teaching resilience and existential recovery.

**Conclusions:**

Fictional case vignettes such as Ash’s can enrich psychiatric education by safely demonstrating complex themes—attachment trauma, identity collapse, resilience and existential recovery—beyond traditional diagnostic frameworks. Integrating creative narratives into training may foster empathy, conceptual flexibility and deeper understanding of patient experiences.

**Disclosure of Interest:**

None Declared

